# Hunting as a management tool? Cougar-human conflict is positively related to trophy hunting

**DOI:** 10.1186/s12898-016-0098-4

**Published:** 2016-10-11

**Authors:** Kristine J. Teichman, Bogdan Cristescu, Chris T. Darimont

**Affiliations:** 1 Department of Geography, University of Victoria, PO Box 3060, STN CSC, Victoria, BC V8W 3R4 Canada; 2 Biology Department, University of British Columbia, 3333 University Way, Kelowna, BC V1V 1V7 Canada; 3 Department of Biological Sciences, University of Cape Town, Private Bag X3, Rondebosch, 7701 South Africa; 4 Raincoast Conservation Foundation, P.O. Box 77, Bella Bella, BC V0T 1B0 Canada; 5 Hakai Institute, P.O. Box 309, Heriot Bay, BC V0P 1H0 Canada

**Keywords:** British Columbia, Mountain lion, Predator-human coexistence, Puma, *Puma concolor*, Skull size, Trophy hunting, Wildlife

## Abstract

**Background:**

Overexploitation and persecution of large carnivores resulting from conflict with humans comprise major causes of declines worldwide. Although little is known about the interplay between these mortality types, hunting of predators remains a common management strategy aimed at reducing predator-human conflict. Emerging theory and data, however, caution that such policy can alter the age structure of populations, triggering increased conflict in which conflict-prone juveniles are involved.

**Results:**

Using a 30-year dataset on human-caused cougar (*Puma concolor*) kills in British Columbia (BC), Canada, we examined relationships between hunter-caused and conflict-associated mortality. Individuals that were killed via conflict with humans were younger than hunted cougars. Accounting for human density and habitat productivity, human hunting pressure during or before the year of conflict comprised the most important variables. Both were associated with increased male cougar-human conflict. Moreover, in each of five regions assessed, conflict was higher with increased human hunting pressure for at least one cougar sex.

**Conclusion:**

Although only providing correlative evidence, such patterns over large geographic and temporal scales suggest that alternative approaches to conflict mitigation might yield more effective outcomes for humans as well as cougar populations and the individuals within populations.

**Electronic supplementary material:**

The online version of this article (doi:10.1186/s12898-016-0098-4) contains supplementary material, which is available to authorized users.

## Background

Exploitation and persecution related to conflict with humans form major causes of predator declines worldwide [1–4]. Killing takes several forms and its ecological and evolutionary effects might be more severe than the number of removed predators suggests [5]. Expansion of human activities into previously undisturbed areas enables increased killing through facilitated human access; roads, cut lines and trails associated with extractive industries facilitate hunting of predators during and/or after resource extraction [6, 7]. As human populations expand, the likelihood of wildlife-human conflict also increases [8].

When conflicts involve large mammalian predators that pose a perceived or real threat to humans and property, a common outcome is the lethal removal of the predator by management agencies or sometimes by land owners, for example in response to predation on livestock [9]. In addition, conflict is often managed through increasing human-caused killing of carnivores, under the premise that human hunting can reduce conflict incidence over depredation or decrease predation on wild ungulates sought by hunters (hereafter, ‘human hunting hypothesis’; e.g., [10–12]).

In the case of predator-human conflict over depredation, Treves and Naughton-Treves [13] suggested that carnivore killing by hunters may actually promote conflict. The process is thought to operate via shifts in age composition to younger age animals, which might depredate more because of higher encounter rates with livestock. This process is thought to occur via the increased mobility of juvenile age classes of carnivores caused by decline in adult male territory tenure [14]. Young individuals become locally more abundant and thereby have increased chance of encountering livestock—and/or young animals might be bolder, more curious or lacking experience in interactions with people [15] or in capturing wild prey effectively [16]. Collectively these factors suggest that younger animals are more conflict-prone (hereafter, ‘young animal hypothesis’). Moreover, hunting, culling or other lethal control targeted at specific individuals (e.g. those involved in livestock predation) may reduce conflict (‘problem individuals hypothesis’; e.g., [16]), which has been challenged by the assertion that dispersing individuals often quickly recolonize conflict areas, offering only temporary relief [17].

To confront these hypotheses, we examined a long-term dataset on human hunting of cougars and conflict involving cougars in BC, Canada. Cougar-human conflict and cougar hunting are relatively widespread and common, the latter attracting both local BC hunters as well as foreign hunters for guided hunts. We used this system to test whether: (1) cougars killed by hunters would be larger than those that came into conflict with people (young animal hypothesis); and (2) human hunting mortality and conflict incidence would be related (problem animal and human hunting hypotheses).

## Methods

### Cougar data

We used a 30-year dataset (1979–2008) on recorded cougar mortality in BC, Canada provided by the BC Ministry of Environment, wherein all records had an associated date. We used cougar kill records resulting from conflict and legal hunting events. For analyses involving age of conflict and legally hunted cougars [(1) above] we used only those records with associated spatial data, sex and skull sizes. The other analyses [(2) above] were carried out using the larger dataset of spatially-referenced conflict and legal hunting mortalities of cougars with known sex, irrespective of whether skull size had been recorded. Only 96 illegal kills were recorded during 1979–2008, of which 35 had associated skull length and width data and these were not used in analyses. We consider this a minimum estimate because evaluations of the frequency of illegal cougar kills have not been performed. We do not expect illegal killing to vary across regions. Additional spatially-referenced mortality records of cougars with known sex (356, of which 139 had associated skull information) had unclear or unrecorded cause of death and were not used in analyses.

Spatial data included universal transverse mercator (UTM) coordinates and we considered only conflict and legal hunting records occurring within the 5 of 8 total ‘development regions’ of BC (region size mean ± SE, 72,173 ± 19,388 km^2^) in which mortality was highest (Cariboo, Kootenay, Lower Mainland South-West (SW), Thompson Okanagan and Vancouver Island). After plotting kill locations by region in ArcGIS v.10.3 (ESRI, Redlands, USA) for validation and discarding records occurring outside the 5 regions or in water, as well as a small number of erroneous records (e.g., skull width > skull length), the final dataset for cougar age analysis consisted of 3665 records. The data included records of kills by BC resident hunters and non-resident guided hunters (*n* = 3219) as well as conflict-related cougar deaths (*n* = 449). ‘Conflict’ was defined as any incident of cougar road mortality, predation on livestock, perceived risk to people such as cougars sighted in urban areas, or recorded attack on humans. More male (*n* = 2240) than female (*n* = 1428) mortality records occurred in the data. The larger dataset for analysis of cougar conflict in relation to human hunting levels included 8788 records. The data were dominated by hunting mortalities (*n* = 7550), with conflict-related kills being less frequent (*n* = 1238). The dataset had more male (*n* = 5348) than female records (*n* = 3440).

Skull size data (length and width in mm) were collected by BC Ministry of Environment personnel as a proxy for age. These variables are positively correlated [18] with the skull growth continuing long into adulthood [19]. Skull size has been used as a proxy for age/body size in other large felids, such as African lion [20], leopard [21] and jaguar [22]. Because skull length and width were highly correlated for males (Pearson *r* = 0.761, *df* = 2239, *P* < 0.001) and females (*r* = 0.669, *df* = 1427, *P* < 0.001), we used an index known as the total skull length (or total skull size) for all analyses. This index is the sum of length and width [22] and is the standard age/body/trophy size metric used by the Boone and Crockett Club and the International Council for Game and Wildlife Conservation when assessing cougar and jaguar trophies [23].

### Statistical analyses

To assess if skull sizes varied in relation to different human-caused mortality types, we first assessed if the variable was normally distributed with Shapiro–Wilk tests. Separate assessments were carried out for each sex and region. For males and females in all regions, the skull size variable was not normally distributed. Therefore we used two-sample Wilcoxon rank-sum (Mann–Whitney) tests to compare mean skull size for conflict and hunter kills. Separate testing was performed for each sex and region for a total of 10 tests (2 sexes × 5 regions).

We used time series analysis to test factor combinations hypothesized a priori to influence annual conflict frequency across time (Additional file 1: Table S1). Newey-West Heteroskedasticity and Autocorrelation (HAC) standard errors were computed in multiple linear regression to account for potential variability and temporal autocorrelation in the models’ error terms. Conflict incidence (dependent variable) was standardized per 10,000 km^2^ and square root-transformed prior to modelling to reduce skewness. Predictor variables included human density (D), human hunting pressure (annual number of cougars hunted) in the year of conflict (H_t0_) and the Normalized Difference Vegetation Index (NDVI; a proxy for plant and prey productivity) in the year of conflict (N_t0_). A squared term was included for human density (D^2^) to account for possible thresholds in human density beyond which conflict would decrease because of an assumed limitation to cougar habitat. Yearly lag 1 and 2 terms were used for human hunting pressure (H_t1_; H_t2_) and NDVI (N_t1_; N_t2_) to incorporate potential influences of hunting and habitat productivity in the periods preceding conflict. Human density (per 10,000 km^2^) was calculated for each year by dividing annual census counts by region size (details in Additional file 2). Human density calculation for the Vancouver Island region included a small part of the mainland coast as constrained by data availability. Human hunting pressure was standardized per 10,000 km^2^ and included hunting by residents and non-residents of BC. Because habitat quality and prey availability can influence large carnivore-human conflict [24, 25], but such data over our broad temporal and spatial extents were not available, we used NDVI as a habitat productivity surrogate [26–28]. These data came from the National Oceanic and Atmospheric Administration (NOAA) Climate Data Records (CDR), which derived NDVI from surface reflectance data acquired by the advanced very high resolution radiometer (AVHRR) sensor ([29]; details in Additional file 2). Highly correlated variables (*r* > |0.8|) were not included together in the same model structure..

We evaluated candidate models using Akaike’s Information Criterion for small sample sizes (AICc) [30]. We estimated relative importance of variables by applying multi-model inference to rank variables in the supported model set (ΔAICc ≤ 7) by their summed AICc weights [31]. We used the proportion of variance explained (*R* ^2^) to evaluate model performance. For all models that received support we plotted residuals against fitted values and inspected for patterns in the residual distribution. We used Stata v.14.1 (StataCorp, College Station, USA) and an alpha level of 0.10 for all statistical analyses. The Newey-West HAC standard errors were computed in Stata using the hacreg command [32].

## Results

### Skull size comparisons between hunter- and conflict-killed cougars

**Fig. 1:**
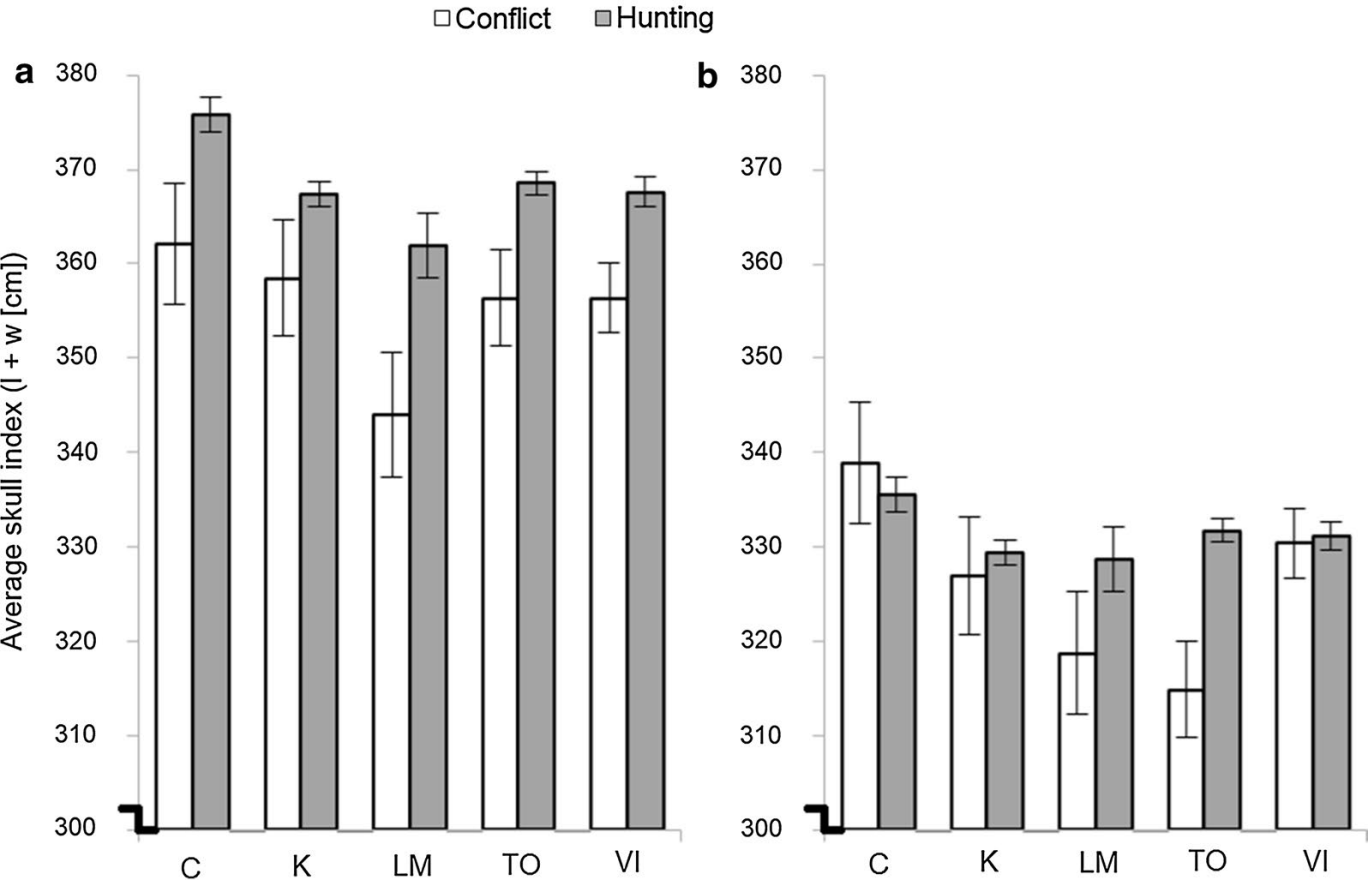
Average skull sizes of cougars killed in five regions of British Columbia, Canada, as a result of conflict and human hunting. Data include kill records with associated geographic coordinates and age (skull size) information for **a** males and **b** females. BC regions are: *C* Cariboo, *K* Kootenay, *LM* Lower Mainland SW, *TO* Thompson Okanagan and *VI* Vancouver Island. *Error bars* represent ± 1 SE. Note broken *Y axis*

At the provincial level, conflict-killed male cougar skulls were smaller than those of hunter-killed animals (Two-sample Wilcoxon rank-sum *z* = −5.376, *df* = 2239, *P* < 0.001). Skull sizes differed between kill types for males in 4 of the 5 BC regions, similarly larger for hunter-killed than for conflict-killed males for Cariboo (Two-sample Wilcoxon rank-sum *z* = −1.959, *df* = 329, *P* = 0.050), Lower Mainland SW (Two-sample Wilcoxon rank-sum *z* = −2.195, *df* = 113, *P* = 0.028), Thompson Okanagan (Two-sample Wilcoxon rank-sum *z* = −2.210, *df* = 763, *P* = 0.027) and Vancouver Island (Two-sample Wilcoxon rank-sum *z* = −2.762, *df* = 571, *P* = 0.006) (Fig. 1a).

At the provincial level, skull sizes of females were similarly smaller among conflict animals compared with hunter-killed individuals (Two-sample Wilcoxon rank-sum *z* = −3.464, *df* = 1427, *P* < 0.001). Skull sizes likewise differed between kill types in 2 of the 5 BC regions (Lower Mainland SW; Two-sample Wilcoxon rank-sum *z* = −1.701, *df* = 114, *P* = 0.089; Thompson Okanagan; Two-sample Wilcoxon rank-sum *z* = −4.311, *df* = 520, *P* < 0.001) (Fig. 1b).

### Predictors of cougar-human conflict

Regional models (see Additional file 3) that received substantial support explained roughly half of the variation in cougar-human conflict for males (*R* ^2^: *mean* = 0.504; *range* = 0.258–0.816; all *P* < 0.10) as well as females (*R* ^2^: *mean* = 0.507; *range* = 0.124–0.772; all *P* < 0.10). For both sexes, models that received substantial support were of intermediate or low complexity (with 1‒4 parameters, including the intercept; Table 1). Only for males in the Lower Mainland SW did the intercept-only model receive substantial support, but two candidate models were superior. All supported models (ΔAICc ≤ 7) [33] are listed in Additional file 4: Tables S2–S6, provided their ΔAICc was smaller than that of the corresponding null model.

**Table 1:** Models for assessing temporal patterns of cougar-human conflict in British Columbia, Canada that received substantial support (ΔAICc < 2)

Region	Sex	Model description	ΔAICc	*w*_AICc_	R^2^
Cariboo	Male	D + D^2^ + H_t0_	0.0	0.33	0.557
		D + D^2^	1.0	0.20	0.456
		H_t0_	1.1	0.19	0.369
	Female	D + D^2^	0.0	0.64	0.599
Kootenay	Male	N_t0_ + H_t0_	0.0	0.82	0.816
	Female	D + D^2^	0.0	0.48	0.736
		N_t0_ + D + D^2^	0.3	0.42	0.772
Lower Mainland SW	Male	H_t1_ + H_t2_	0.0	0.19	0.258
		H_t0_ + H_t1_ + H_t2_	0.6	0.14	0.347
	Female	D + D^2^	0.0	0.46	0.334
Thompson Okanagan	Male	D + D^2^ + H_t0_	0.0	0.51	0.590
	Female	H_t0_ + H_t1_ + H_t2_	0.0	0.30	0.406
		H_t0_	1.8	0.12	0.124
		D + D^2^	1.8	0.12	0.236
Vancouver Island	Male	H_t1_ + H_t2_	0.0	0.35	0.539
		H_t0_ + H_t1_ + H_t2_	0.2	0.32	0.602
	Female	H_t0_	0.0	0.50	0.668
		N_t0_ + H_t0_	1.6	0.23	0.688

*D* human density, *H*_*t0*_ human hunting pressure, *H*_*t1*_ Human hunting pressure (lag 1), *H*_*t2*_ human hunting pressure (lag 2), *N*_*t0*_ NDVI, *N*_*t1*_ NDVI (lag 1), *N*_*t2*_ NDVI (lag 2)

**Fig. 2:**
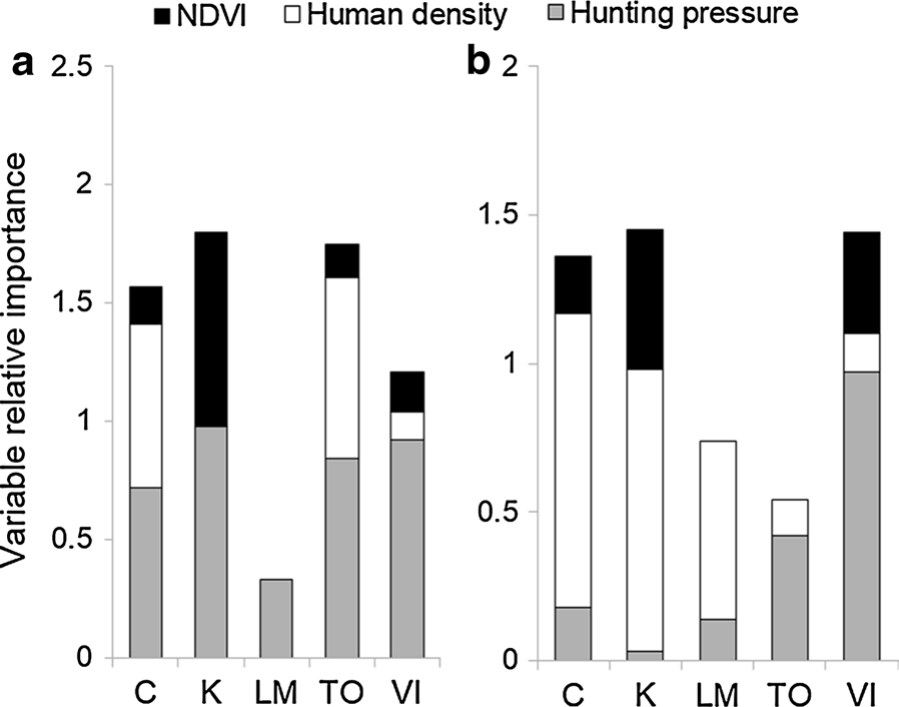
Relative importance of variables associated with cougar-human conflict in British Columbia, Canada for **a** males and **b** females. Importance values were calculated by summing AICc weights of models that included the respective variable and which received support (ΔAICc ≤ 7). *C* Cariboo, *K* Kootenay, *LM* Lower Mainland SW, *TO* Thompson Okanagan and *VI* Vancouver Island

**Fig. 3:**
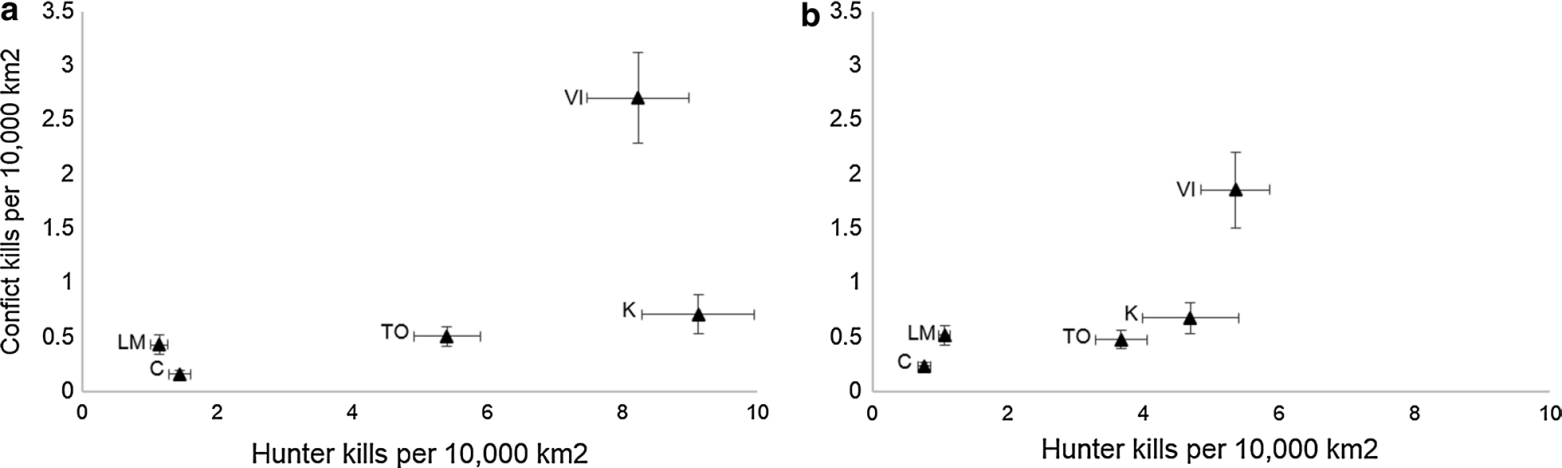
Mean (±1 SE) annual conflict-killed relative to hunter-killed cougars per 10,000 km^2^ in five regions of British Columbia, Canada. Data are for **a** males and **b** females. *C* Cariboo, *K* Kootenay, *LM* Lower Mainland SW, *TO* Thompson Okanagan and *VI* Vancouver Island

Human hunting pressure in both current (Figs. 2, 3) and lagged periods (Fig. 2) had the most relative importance for predicting cougar-human conflict for male cougars across the five regions. Human hunting was positively associated with conflict involving this cougar sex. Variables for human hunting during the conflict year or hunting lagged occurred in all but one male model that received substantial support and which had AICc less than the null model’s AICc (Table 1).

Human hunting pressure was also the most important factor associated with cougar-human conflict for female cougars in 2 of 5 BC regions. Only for one model (female cougars, Thompson-Okanagan) was increased human hunting (lag 2) associated with decreased conflict.

Overall, increased human hunting was related to greater conflict for 16 of 17 models that included hunting variables with estimates that did not overlap zero and that received substantial support (Table 2; Additional file 4: Tables S7–S11).

**Table 2:** Direction (+ positive, − negative) and confidence interval overlap with zero for parameter estimates from substantially supported ΔAICc models for cougar-human conflict in British Columbia, Canada

Region	Sex	N_t0_	N_t1_	N_t2_	D	D^2^	H_t0_	H_t1_	H_t2_
Cariboo	Male				+ +	‒ ‒	+* +*		
	Female				+	‒			
Kootenay	Male	‒*					+*		
	Female	‒*			‒*‒*	+* + *			
Lower Mainland SW	Male						+*	+* + *	‒ ‒
	Female				+*	‒*			
Thompson Okanagan	Male				+*	‒*	+*		
	Female				+*	‒*	+* + *	‒	‒*
Vancouver Island	Male						+*	+* + *	+* + *
	Female	‒*					+* + *		

Estimates for which confidence intervals did not overlap zero have an asterisk. No reporting of coefficients refers to the specific variable(s) not being included in supported models

*D* human density, *H*_*t0*_ human hunting pressure, *H*_*t1*_ Human hunting pressure (lag 1), *H*_*t2*_ human hunting pressure (lag 2), *N*_*t0*_ NDVI, *N*_*t1*_ NDVI (lag 1), *N*_*t2*_ NDVI (lag 2)

Human density was the key variable associated with conflict for female cougars in 3 BC regions (Fig. 2) and was also important for male cougar-human conflict in 1 BC region (Table 2). Years with intermediary human densities were generally associated with conflict (Additional file 4: Tables S9, S10). For both cougar sexes, NDVI was the least important variable tested in relation to conflict (Fig. 2), but three substantially supported models revealed conflict increases in years when habitat productivity was low (Table 2).

## Discussion

With expanding human populations and influence, conflict between carnivores and humans is expected to increase, which requires evidence-informed approaches to conflict mitigation. A long-term data set on human-caused cougar mortality allowed us to confront fundamental hypotheses on the relationship between human hunting, cougar-human conflict and cougar population demography, including testing of the commonly accepted but under-examined assumption that hunting of large carnivores could result in decreased conflict incidence (see [34] for an overview and call for inquiry into the relationship between hunting of carnivores and conflict).

As we expected, we found support for the young animal hypothesis in most comparisons, with individuals that came into conflict with humans younger compared to those hunted. Human encroachment into cougar habitat increases conflict potential [35–37] and young animals are more likely to occur in areas used by people than other age classes [38]. Dispersing juveniles are more likely to come into conflict on travel routes through fragmented habitats and high risk areas including human inhabited areas, roads [39] and ranches [24, 40]. In addition, food resources may be limited while dispersers attempt to establish home ranges [41]. As a result, when available, cougars may attack livestock [42] (however, see [43] for an alternative documentation of old cougars being disproportionately involved in livestock predation). Finally, hunters might be more likely to forgo killing small individuals for trophies, particularly if they are treed by trained hounds, although this has not been examined.

The manner by which carnivore populations respond to regulated hunting depends on social structure, reproductive strategies and dispersal patterns [14]. Human hunting of old individuals can increase immigration of juveniles from neighboring areas [14, 44], which could result in increased conflict. We therefore hypothesized that increased human hunting pressure would be associated with increased conflict via social disruption and younger population age structure (problem animal and human hunting hypotheses). We demonstrated that high hunting-related mortality in the same or preceding time period is positively associated with cougar-human conflict for at least one sex in all five regions tested (Table 2; Figs. 2, 3), with the most consistent pattern (both sexes: regression *P* < 0.10) for Thompson-Okanagan and Vancouver Island. While Thompson-Okanagan is an inland region, Vancouver Island is a large land mass off the British Columbia mainland known to be the world’s ‘hotspot’ of cougar-human conflict [45]. Our results corroborate and extend recent findings on impacts of human hunting on cougar complaints and depredations in Washington State [46]. In British Columbia, male cougars appeared most susceptible to conflict if hunted more intensively and conflict records involving males were almost double in number than those involving females. The latter findings are in accordance with Linnell et al.’s conclusion that male large carnivores are most likely to get into conflict with humans [16], a proposition also more recently supported by research on cheetah-human [47] and jaguar-human conflicts [48]. One mechanism that might explain why males of hunted cougar populations are involved more frequently in conflicts than females might be the altered male spatial organization under greater hunting pressure [49].

Human densities were associated with male cougar-human conflict in only one BC region, whereas conflict with females appeared related to variation in human density. Females might use suboptimal areas with human development by means of spatially avoiding male-caused mortality risk for themselves and their offspring, possibly resulting in increased conflict for females in connection to human densities, as we detected. Selection of areas close to human development by females with offspring presumably to avoid males has been recently documented for cougars in California [50] and grizzly bears in Alberta [51]. Thompson-Okanagan was the only region where human density was related with conflict for both sexes, with conflicts most likely at intermediary densities of people. Such intermediate densities are typically found in exurban or suburban areas and are thought to have high levels of cougar-human conflict in California [52]. Despite high human populations in Lower Mainland SW, human density in this region did not influence frequency of conflict involving males. The documented decreases in conflict associated with decreased human hunting of males in this region suggest that, similar to other carnivores [53], cougar populations can persist in regions with high human densities as long as human hunting pressure is low.

We found limited relationship with NDVI, our proxy for habitat productivity. Decreased productivity was hypothesized to be associated with increased cougar-human conflict. Conversely, a positive relation between conflict and NDVI might have been expected due to increased productivity resulting in increased reproductive output [54], with the indirect effect of increased sub-adult dispersal and greater conflict potential. Kootenay was the only region where decreased productivity was associated with increased conflict for both males and females. This region comprises substantial high elevation mountain ranges compared to the other regions and habitat productivity in the Kootenay is possibly an important limiting factor for cougars and their prey. Future monitoring of the associations between habitat productivity and carnivore-human conflict should not be neglected, given increased variability in vegetation conditions/NDVI associated with climate change, which might have implications for future predator-human conflicts that have yet to be explored. When possible, finer scale prey availability metrics should be incorporated, because prey use differences among cougar sexes [55] could influence conflict incidence. Furthermore, it is important to recognize that inferences from this study should be placed in the context of the relative coarseness of covariate data utilized, which is to be expected when focusing on broad spatiotemporal extents such as the one we considered. Our results showed that human-related variables had the strongest association with conflict. We acknowledge that the patterns of association we reveal do not necessarily imply causation. Our results, however, are generally consistent with the hypothesis that high hunter mortality leads to young animals becoming involved in conflict. Unlike natural agents of mortality (other predators, competitors, disease), hunters typically target adult individuals. The ability of resident males to maintain territories means that sub-adults are more likely to come into conflict, likely because of their movements during dispersal in search for vacant territories [56]. Human hunting can disrupt social structure leading to increased juvenile immigration from surrounding source populations [14] and result in younger age structure [57, 58] exacerbating conflicts between cougars and humans. With increasing human populations, interactions between predators and humans are expected to become more common, underlining the need for research into patterns and mechanisms of conflict, conflict prevention and non-traditional management strategies to facilitate coexistence.

## Conclusions

Wildlife managers often prescribe hunting of carnivores to reduce competition with hunters for prey and to minimize conflicts with humans and their property [8]. If lethal control such as through human hunting is to facilitate coexistence between wildlife and humans, control must minimize wildlife-human conflict or increase tolerance of the public towards wildlife, without compromising wildlife population viability [13]. In some situations lethal management focused on targeted individuals associated with conflict (e.g., individuals that injure or kill people in predatory attacks) offers one route to address large carnivore-human conflicts. However, we showed that overall increased human hunting in fact can be associated with increased conflict, especially for males. Although our results are only correlative, we caution against the universal use of hunting as a tool for managing conflict with large predators.

## Additional files

10.1186/s12898-016-0098-4 Hypotheses for frequency of cougar-human conflict.

10.1186/s12898-016-0098-4 Information on NDVI and human density data.

10.1186/s12898-016-0098-4 Information on regional models of cougar-human conflict.

10.1186/s12898-016-0098-4 List of all cougar-human conflict models that received support (ΔAICc ≤ 7). Estimated coefficients from substantially supported (ΔAICc < 2) models are also listed.

## Abbreviations

AICc: Akaike’s information criterion for small sample sizes
AVHRR: advanced very high resolution radiometer
BC: British Columbia
CDR: climate data records
D: human density
HAC: heteroskedasticity and autocorrelation
H_t0_: human hunting pressure
H_t1_: human hunting pressure (lag 1)
H_t2_: human hunting pressure (lag 2)
NDVI: normalized difference vegetation index
NOAA: national oceanic and atmospheric administration
N_t0_: NDVI
N_t1_: NDVI (lag 1)
N_t2_: NDVI (lag 2)
SW: south–west
UTM: universal transverse mercator
ΔAICc: difference between the model AICc and the lowest AICc for the model set
