# Supplementary material for: Hunting as a management tool? Cougar-human conflict is positively related to trophy hunting
Source: BMC Ecol. 2016 Oct 11;16:44. doi: 10.1186/s12898-016-0098-4 (PMC5057462; doi:10.1186/s12898-016-0098-4)
Supplement: Supplementary file 1 — 10.1186/s12898-016-0098-4 Hypotheses for frequency of cougar-human conflict. [file 12898_2016_98_MOESM1_ESM.docx]

**Additional file 1: Table S1.**

Teichman KJ, Cristescu B, Darimont CT. Hunting as a management tool? Cougar-human conflict is positively related to trophy hunting. BMC Ecology.

**Table S1** Hypotheses for frequency of cougar-human conflict

| **Hypothesis** | **Model description** |
| --- | --- |
| Current habitat state | N_t0_ |
| Previous habitat state | N_t1_ +N_t2_ |
| Human density | D + D^2^ |
| Current habitat state + Human density | N_t0_ + D + D^2^ |
| Previous habitat state + Human density | N_t1_ + N_t2_ + D + D^2^ |
| Current human hunting pressure | H_t0_ |
| Previous human hunting pressure | H_t1_ + H_t2_ |
| Current & Previous human hunting pressure | H_t0_ + H_t1_ + H_t2_ |
| Human density + Current human hunting pressure | D + D^2^ + H_t0_ |
| Human density + Previous human hunting pressure | D + D^2^ + H_t1_ + H_t2_ |
| Human density + Current & Previous human hunting pressure | D + D^2^ + H_t0_ + H_t1_ + H_t2_ |
| Current habitat state + Current human hunting pressure | N_t0_ + H_t0_ |
| Previous habitat state + Current human hunting pressure | N_t1_ + N_t2_ + H_t0_ |
| Previous habitat state + Previous human hunting pressure | N_t1_ + N_t2_ + H_t1_ + H_t2_ |
| Current habitat state + Current & Previous human hunting pressure | N_t0_ + H_t0_ + H_t1_ + H_t2_ |
| Previous habitat state + Current & Previous human hunting pressure | N_t1_ + N_t2_ + H_t0_ + H_t1_ + H_t2_ |
| Current habitat state + Human density + Current human hunting pressure | N_t0_ + D + D^2^ + H_t0_ |
| Previous habitat state + Human density + Previous human hunting pressure | N_t1_ + N_t2_ + D + D^2^ + H_t1_ + H_t2_ |
| Current habitat state + Human density + Current & Previous human hunting pressure | N_t0_ + D + D^2^ + H_t0_ + H_t1_ + H_t2_ |
| Previous habitat state + Human density + Current & Previous human hunting pressure | N_t1_ + N_t2_ + D + D^2^ + H_t0_ + H_t1_ + H_t2_ |

D - Human density; H_t0_ - Human hunting pressure; H_t1_ – Human hunting pressure (lag 1); H_t2_ – Human hunting pressure (lag 2); N_t0_ – NDVI; N_t1_ – NDVI (lag 1); N_t2_ – NDVI (lag 2)
