# Supplementary material for: Hunting as a management tool? Cougar-human conflict is positively related to trophy hunting
Source: BMC Ecol. 2016 Oct 11;16:44. doi: 10.1186/s12898-016-0098-4 (PMC5057462; doi:10.1186/s12898-016-0098-4)
Supplement: Supplementary file 2 — 10.1186/s12898-016-0098-4 Information on NDVI and human density data. [file 12898_2016_98_MOESM2_ESM.docx]

**Additional file 2: Text S1.**

Teichman KJ, Cristescu B, Darimont CT. Hunting as a management tool? Cougar-human conflict is positively related to trophy hunting. BMC Ecology.

**Information on NDVI and human density data**

**NDVI data**

Normalized Difference Vegetation Index (NDVI) data (NOAA CDR NDVI) were downloaded via the EarthExplorer web portal of United States Geological Survey (http://earthexplorer.usgs.gov/). Although NDVI data were available as early as 1981, only 1988-2007 data were used as constrained by human and cougar population data availability as well as by the inclusion of lagged covariates (see Additional file 3: Text S2).

For each study year three NDVI datasets were downloaded (June 14, 21 and 28). We initially attempted to also incorporate June 7 data but were unable to do so because June 7 NDVI did not cover the study area extent during one of the study years. The June 14, 21 and 28 rasters (cell size 5.34 km × 5.34 km) were summed in ArcGIS 10.3 using MapAlgebra (e.g., [1]) and the resulting raster was clipped to study area extent. Raster values were then extracted for each year and each BC region and the mean for each region was standardized by dividing it by the region’s land area.

**Human density data**

Data on human population sizes for each BC development region were extracted from BC population census records which were available annually since 1986 (http://www.bcstats.gov.bc.ca/StatisticsBySubject/Demography/PopulationEstimates.aspx). Human density was calculated by dividing the total population for a given region by the region’s land area.

1. Pettorelli N, Pelletier F, von Hardenberg A, Festa-Bianchet M, Côté SD. Early onset of vegetation growth vs. rapid green-up: impacts on juvenile mountain ungulates. Ecology. 2007;88:381–390.
