# Supplementary material for: Hunting as a management tool? Cougar-human conflict is positively related to trophy hunting
Source: BMC Ecol. 2016 Oct 11;16:44. doi: 10.1186/s12898-016-0098-4 (PMC5057462; doi:10.1186/s12898-016-0098-4)
Supplement: Supplementary file 3 — 10.1186/s12898-016-0098-4 Information on regional models of cougar-human conflict. [file 12898_2016_98_MOESM3_ESM.docx]

**Additional file 3: Text S2.**

Teichman KJ, Cristescu B, Darimont CT. Hunting as a management tool? Cougar-human conflict is positively related to trophy hunting. BMC Ecology.

**Information on regional models of cougar-human conflict**

The dataset for regional cougar-human conflict modelling included data with and without associated skull size in order to provide a comprehensive representation of conflict and hunting frequency in British Columbia. The time frame of conflict modelling was shorter (20 years: 1988-2007) than the time frame of the cougar age analysis (30 years: 1979-2008). The reasons for the shorter time frame for conflict modelling were: 1. Availability of human population census data (no data prior to 1986); 2. Inability to use years 1986 and 1987 because of lagged covariates (up to lag 2, wherein lags were annual); 3. Dropping of year 2008 because no mortality records were available past March 2008 (use of January – March 2008 records would have underestimated hunting and conflict frequency for the respective year).

Human hunting (H_t0_) and hunting pressure lag 1 (H_t1_), as well as hunting pressure lag 1(H_t1_) and hunting pressure lag 2 (H_t2_) were highly correlated for Kootenay male and female cougars. Human hunting (H_t0_), hunting lag 1 (H_t1_) and hunting lag 2 (H_t2_) were each highly correlated with human density for Kootenay males. Hunting (H_t0_) only was highly correlated with human density for Kootenay females. All coefficients for these strong correlations were positive. Excluding models with these variable combinations resulted in 2 sets of candidate models (Kootenay male; Kootenay female) that only included 8 models each. Model sets for all other BC region-sex pairs included 20 candidate models (Additional file 1: Table S1).

For each region and sex we report in the main text the models that received substantial support (ΔAICc <2) (Table 1) as well as direction (sign) of the respective parameter estimates and whether their confidence intervals overlapped zero (Table 2). We include in the supplementary information the actual parameter values for substantially supported models (Additional file 4: Tables S7-S11). For supported models we did not detect curvature or increasing/decreasing variation in the residuals that could have been indicative of violation of least-squares assumptions.
