# Supplementary material for: Hunting as a management tool? Cougar-human conflict is positively related to trophy hunting
Source: BMC Ecol. 2016 Oct 11;16:44. doi: 10.1186/s12898-016-0098-4 (PMC5057462; doi:10.1186/s12898-016-0098-4)
Supplement: Supplementary file 4 — 10.1186/s12898-016-0098-4 List of all cougar-human conflict models that received support (ΔAICc ≤ 7). Estimated coefficients from substantially supported (ΔAICc < 2) models are also listed. [file 12898_2016_98_MOESM4_ESM.docx]

**Additional file 4: Tables S2-S11.**

Teichman KJ, Cristescu B, Darimont CT. Hunting as a management tool? Cougar-human conflict is positively related to trophy hunting. BMC Ecology.

**Table S2** Supported models (ΔAICc ≤7) for temporal patterns of cougar-human conflict in the Cariboo region, British Columbia

| **Cougar sex** | **Model description** | **ΔAICc** | ***w*_AICc_** | **R^2^** |
| --- | --- | --- | --- | --- |
| Male | D + D^2^ + H_t0_ | 0.0 | 0.33 | 0.557 |
|  | D + D^2^ | 1.0 | 0.20 | 0.456 |
|  | H_t0_ | 1.1 | 0.19 | 0.369 |
|  | N_t0_ + D + D^2^ + H_t0_ | 3.3 | 0.06 | 0.564 |
|  | N_t0_ + H_t0_ | 3.6 | 0.05 | 0.380 |
|  | N_t0_ + D + D^2^ | 4.1 | 0.04 | 0.456 |
|  | D + D^2^ + H_t0_ + H_t1_ + H_t2_ | 4.4 | 0.04 | 0.626 |
|  | H_t0_ + H_t1_ + H_t2_ | 5.5 | 0.02 | 0.418 |
|  | D + D^2^ + H_t1_ + H_t2_ | 5.8 | 0.02 | 0.507 |
|  | N_t1_ + N_t2_ + H_t0_ | 6.7 | 0.01 | 0.382 |
| Female | D + D^2^ | 0.0 | 0.64 | 0.599 |
|  | N_t0_ + D + D^2^ | 3.1 | 0.14 | 0.600 |
|  | D + D^2^ + H_t0_ | 3.2 | 0.13 | 0.599 |
|  | N_t1_ + N_t2_ + D + D^2^ | 6.0 | 0.03 | 0.615 |
|  | D + D^2^ + H_t1_ + H_t2_ | 6.3 | 0.03 | 0.609 |
|  | N_t0_ + D + D^2^ + H_t0_ | 6.7 | 0.02 | 0.600 |

D - Human density; H_t0_ – Human hunting pressure; H_t1_ – Human hunting pressure (lag 1); H_t2_ – Human hunting pressure (lag 2); N_t0_ – NDVI; N_t1_ - NDVI (lag 1); N_t2_ - NDVI (lag 2)

**Table S3** Supported models (ΔAICc ≤7) for temporal patterns of cougar-human conflict in the Kootenay region, British Columbia

| **Cougar sex** | **Model description** | **ΔAICc** | ***w*_AICc_** | **R^2^** |
| --- | --- | --- | --- | --- |
| Male | N_t0_ + H_t0_ | 0.0 | 0.82 | 0.816 |
|  | H_t0_ | 3.2 | 0.16 | 0.752 |
| Female | D + D^2^ | 0.0 | 0.48 | 0.736 |
|  | N_t0_ + D + D^2^ | 0.3 | 0.42 | 0.772 |
|  | N_t1_ + N_t2_ + D + D^2^ | 4.4 | 0.05 | 0.765 |
|  | H_t0_ | 5.5 | 0.03 | 0.601 |

D - Human density; H_t0_ – Human hunting pressure; H_t1_ – Human hunting pressure (lag 1); H_t2_ – Human hunting pressure (lag 2); N_t0_ – NDVI; N_t1_ - NDVI (lag 1); N_t2_ - NDVI (lag 2)

**Table S4** Supported models (ΔAICc ≤7) for temporal patterns of cougar-human conflict in the Lower Mainland SW region, British Columbia

| **Cougar sex** | **Model description** | **ΔAICc** | ***w*_AICc_** | **R^2^** |
| --- | --- | --- | --- | --- |
| Male | H_t1_ + H_t2_ | 0.0 | 0.19 | 0.258 |
|  | H_t0_ + H_t1_ + H_t2_ | 0.6 | 0.14 | 0.347 |
|  | Null | 0.7 | 0.13 | 0.000 |
| Female | D + D^2^ | 0.0 | 0.46 | 0.334 |
|  | D + D^2^ + H_t0_ | 2.3 | 0.14 | 0.362 |
|  | Null | 2.9 | 0.11 | 0.000 |

D - Human density; H_t0_ – Human hunting pressure; H_t1_ – Human hunting pressure (lag 1); H_t2_ – Human hunting pressure (lag 2)

**Table S5** Supported models (ΔAICc ≤7) for temporal patterns of cougar-human conflict in the Thompson-Okanagan region, British Columbia

| **Cougar sex** | **Model description** | **ΔAICc** | ***w*_AICc_** | **R^2^** |
| --- | --- | --- | --- | --- |
| Male | D + D^2^ + H_t0_ | 0.0 | 0.51 | 0.590 |
|  | H_t0_ | 3.4 | 0.09 | 0.345 |
|  | N_t0_ + D + D^2^ + H_t0_ | 3.6 | 0.08 | 0.590 |
|  | D + D^2^ + H_t0_ + H_t1_ + H_t2_ | 3.9 | 0.07 | 0.663 |
|  | D + D^2^ | 4.1 | 0.06 | 0.409 |
|  | D + D^2^ + H_t1_ + H_t2_ | 5.4 | 0.03 | 0.551 |
|  | N_t0_ + H_t0_ | 6.1 | 0.02 | 0.349 |
|  | N_t0_ + D + D^2^ | 6.2 | 0.02 | 0.440 |
|  | N_t1_ + N_t2_ + H_t0_ | 6.4 | 0.02 | 0.435 |
|  | H_t0_ + H_t1_ + H_t2_ | 6.4 | 0.02 | 0.434 |
| Female | H_t0_ + H_t1_ + H_t2_ | 0.0 | 0.30 | 0.406 |
|  | H_t0_ | 1.8 | 0.12 | 0.124 |
|  | D + D^2^ | 1.8 | 0.12 | 0.236 |
|  | Null | 2.0 | 0.11 | 0.000 |

D - Human density; H_t0_ – Human hunting pressure; H_t1_ – Human hunting pressure (lag 1); H_t2_ – Human hunting pressure (lag 2); N_t0_ – NDVI; N_t1_ - NDVI (lag 1); N_t2_ - NDVI (lag 2)

**Table S6** Supported models (ΔAICc ≤7) for tempral patterns of cougar-human conflict in the Vancouver Island region, British Columbia

| **Cougar sex** | **Model description** | **ΔAICc** | ***w*_AICc_** | **R^2^** |
| --- | --- | --- | --- | --- |
| Male | H_t1_ + H_t2_ | 0.0 | 0.35 | 0.539 |
|  | H_t0_ + H_t1_ + H_t2_ | 0.2 | 0.32 | 0.602 |
|  | N_t0_ + H_t0_ + H_t1_ + H_t2_ | 2.0 | 0.13 | 0.637 |
|  | D + D^2^ | 4.1 | 0.04 | 0.433 |
|  | N_t1_ + N_t2_ + H_t1_ + H_t2_ | 4.4 | 0.04 | 0.591 |
|  | D + D^2^ + H_t1_ + H_t2_ | 4.5 | 0.04 | 0.589 |
|  | D + D^2^ + H_t0_ | 4.9 | 0.03 | 0.496 |
|  | D + D^2^ + H_t0_ + H_t1_ + H_t2_ | 6.6 | 0.01 | 0.628 |
| Female | H_t0_ | 0.0 | 0.50 | 0.668 |
|  | N_t0_ + H_t0_ | 1.6 | 0.23 | 0.688 |
|  | D + D^2^ + H_t0_ | 3.2 | 0.10 | 0.711 |
|  | N_t1_ + N_t2_ + H_t0_ | 3.8 | 0.08 | 0.702 |
|  | H_t0_ + H_t1_ + H_t2_ | 5.4 | 0.03 | 0.677 |
|  | N_t0_ + D + D^2^ + H_t0_ | 5.5 | 0.03 | 0.728 |

D - Human density; H_t0_ – Human hunting pressure; H_t1_ – Human hunting pressure (lag 1); H_t2_ – Human hunting pressure (lag 2); N_t0_ – NDVI; N_t1_ - NDVI (lag 1); N_t2_ - NDVI (lag 2)

**Table S7** Estimated coefficients from substantially supported (ΔAICc <2) models to explain cougar-human conflict in the Cariboo region, British Columbia as a function of predictor variables. Variables for which estimates did not overlap zero are given in bold.

| **Cougar sex** | **Variable** | ***β*** | **90% CI** |  |  | ***β*** | **90% CI** |  |  | ***β*** | **90% CI** |  |
| --- | --- | --- | --- | --- | --- | --- | --- | --- | --- | --- | --- | --- |
| Male | Intercept | **-46.10035** | **-92.14958** | **-0.05111** |  | 0.10143 | -0.02686 | 0.22971 |  | -21.82301 | -46.64946 | 3.00344 |
|  | N_t0_ |  |  |  |  |  |  |  |  |  |  |  |
|  | N_t1_ |  |  |  |  |  |  |  |  |  |  |  |
|  | N_t2_ |  |  |  |  |  |  |  |  |  |  |  |
|  | D | 0.00738 | -0.00010 | 0.01488 |  |  |  |  |  | 0.00342 | -0.00062 | 0.00745 |
|  | D^2^ | -2.92e-07 | -5.97e-07 | 1.26e-08 |  |  |  |  |  | -1.32e-07 | -2.95e-07 | 3.18e-08 |
|  | H_t0_ |  |  |  |  | **0.17250** | **0.11631** | **0.22869** |  | **0.10700** | **0.05393** | **0.16008** |
|  | H_t1_ |  |  |  |  |  |  |  |  |  |  |  |
|  | H_t2_ |  |  |  |  |  |  |  |  |  |  |  |
| Female | Intercept | -14.66861 | -45.96336 | 16.62614 |  |  |  |  |  |  |  |  |
|  | N_t0_ |  |  |  |  |  |  |  |  |  |  |  |
|  | N_t1_ |  |  |  |  |  |  |  |  |  |  |  |
|  | N_t2_ |  |  |  |  |  |  |  |  |  |  |  |
|  | D | 0.00211 | -0.00298 | 0.00720 |  |  |  |  |  |  |  |  |
|  | D^2^ | -7.06e-08 | -2.77e-07 | 1.36e-07 |  |  |  |  |  |  |  |  |
|  | H_t0_ |  |  |  |  |  |  |  |  |  |  |  |
|  | H_t1_ |  |  |  |  |  |  |  |  |  |  |  |
|  | H_t2_ |  |  |  |  |  |  |  |  |  |  |  |

D - Human density; H_t0_ – Human hunting pressure; H_t1_ – Human hunting pressure (lag 1); H_t2_ – Human hunting pressure (lag 2); N_t0_ – NDVI; N_t1_ - NDVI (lag 1); N_t2_ - NDVI (lag 2)

**Table S8** Estimated coefficients from substantially supported (ΔAICc <2) models to explain cougar-human conflict in the Kootenay region, British Columbia as a function of predictor variables. Variables for which estimates did not overlap zero are given in bold.

| **Cougar sex** | **Variable** | ***β*** | **90% CI** |  |  | ***β*** | **90% CI** |  |  | ***β*** | **90% CI** |  |
| --- | --- | --- | --- | --- | --- | --- | --- | --- | --- | --- | --- | --- |
| Male | Intercept | **-0.27812** | **-0.33405** | **-0.22219** |  |  |  |  |  |  |  |  |
|  | N_t0_ | **-0.00008** | **-0.00008** | **-0.00007** |  |  |  |  |  |  |  |  |
|  | N_t1_ |  |  |  |  |  |  |  |  |  |  |  |
|  | N_t2_ |  |  |  |  |  |  |  |  |  |  |  |
|  | D |  |  |  |  |  |  |  |  |  |  |  |
|  | D^2^ |  |  |  |  |  |  |  |  |  |  |  |
|  | H_t0_ | **0.12579** | **0.12398** | **0.12761** |  |  |  |  |  |  |  |  |
|  | H_t1_ |  |  |  |  |  |  |  |  |  |  |  |
|  | H_t2_ |  |  |  |  |  |  |  |  |  |  |  |
| Female | Intercept | **36.28935** | **21.92477** | **50.65393** |  | **19.47295** | **6.54181** | **32.40409** |  |  |  |  |
|  | N_t0_ |  |  |  |  | **-0.00005** | **-0.00005** | **-0.00004** |  |  |  |  |
|  | N_t1_ |  |  |  |  |  |  |  |  |  |  |  |
|  | N_t2_ |  |  |  |  |  |  |  |  |  |  |  |
|  | D | **-0.00338** | **-0.00457** | **-0.00219** |  | **-0.00198** | **-0.00305** | **-0.00091** |  |  |  |  |
|  | D^2^ | **7.90e-08** | **5.44e-08** | **1.04e-07** |  | **5.03e-08** | **2.82e-08** | **7.24e-08** |  |  |  |  |
|  | H_t0_ |  |  |  |  |  |  |  |  |  |  |  |
|  | H_t1_ |  |  |  |  |  |  |  |  |  |  |  |
|  | H_t2_ |  |  |  |  |  |  |  |  |  |  |  |

D - Human density; H_t0_ – Human hunting pressure; H_t1_ – Human hunting pressure (lag 1); H_t2_ – Human hunting pressure (lag 2); N_t0_ – NDVI; N_t1_ - NDVI (lag 1); N_t2_ - NDVI (lag 2)

**Table S9** Estimated coefficients from substantially supported (ΔAICc <2) models to explain cougar-human conflict in the Lower Mainland SW region, British Columbia as a function of predictor variables. Variables for which estimates did not overlap zero are given in bold.

| **Cougar sex** | **Variable** | ***β*** | **90% CI** |  |  | ***β*** | **90% CI** |  |  | ***β*** | **90% CI** |  |
| --- | --- | --- | --- | --- | --- | --- | --- | --- | --- | --- | --- | --- |
| Male | Intercept | **0.47228** | **0.29882** | **0.64575** |  | 0.24324 | -0.06011 | 0.54660 |  | -0.11023 | -0.42106 | 0.20060 |
|  | N_t0_ |  |  |  |  |  |  |  |  |  |  |  |
|  | N_t1_ |  |  |  |  |  |  |  |  |  |  |  |
|  | N_t2_ |  |  |  |  |  |  |  |  |  |  |  |
|  | D |  |  |  |  |  |  |  |  |  |  |  |
|  | D^2^ |  |  |  |  |  |  |  |  |  |  |  |
|  | H_t0_ |  |  |  |  |  |  |  |  | **0.19651** | **0.13334** | **0.25968** |
|  | H_t1_ |  |  |  |  | **0.28613** | **0.21799** | **0.35427** |  | **0.34524** | **0.29599** | **0.39448** |
|  | H_t2_ |  |  |  |  | -0.09571 | -0.25288 | 0.06146 |  | -0.06221 | -0.19030 | 0.06588 |
| Female | Intercept | **-6.69909** | **-12.30148** | **-1.09671** |  |  |  |  |  |  |  |  |
|  | N_t0_ |  |  |  |  |  |  |  |  |  |  |  |
|  | N_t1_ |  |  |  |  |  |  |  |  |  |  |  |
|  | N_t2_ |  |  |  |  |  |  |  |  |  |  |  |
|  | D | **0.00003** | **8.35e-06** | **0.00005** |  |  |  |  |  |  |  |  |
|  | D^2^ | **-2.67e-11** | **-4.41e-11** | **-9.34e-12** |  |  |  |  |  |  |  |  |
|  | H_t0_ |  |  |  |  |  |  |  |  |  |  |  |
|  | H_t1_ |  |  |  |  |  |  |  |  |  |  |  |
|  | H_t2_ |  |  |  |  |  |  |  |  |  |  |  |

D - Human density; H_t0_ – Human hunting pressure; H_t1_ – Human hunting pressure (lag 1); H_t2_ – Human hunting pressure (lag 2); N_t0_ – NDVI; N_t1_ - NDVI (lag 1); N_t2_ - NDVI (lag 2)

**Table S10** Estimated coefficients from substantially supported (ΔAICc <2) models to explain cougar-human conflict in the Thompson-Okanagan region, British Columbia as a function of predictor variables. Variables for which estimates did not overlap zero are given in bold.

| **Cougar sex** | **Variable** | ***β*** | **90% CI** |  |  | ***β*** | **90% CI** |  |  | ***β*** | **90% CI** |  |
| --- | --- | --- | --- | --- | --- | --- | --- | --- | --- | --- | --- | --- |
| Male | Intercept | **-20.34270** | **-26.83561** | **-13.84979** |  |  |  |  |  |  |  |  |
|  | N_t0_ |  |  |  |  |  |  |  |  |  |  |  |
|  | N_t1_ |  |  |  |  |  |  |  |  |  |  |  |
|  | N_t2_ |  |  |  |  |  |  |  |  |  |  |  |
|  | D | **0.00094** | **0.00065** | **0.00124** |  |  |  |  |  |  |  |  |
|  | D^2^ | **-1.07e-08** | **-1.39e-08** | **-7.53e-09** |  |  |  |  |  |  |  |  |
|  | H_t0_ | **0.08910** | **0.05213** | **0.12608** |  |  |  |  |  |  |  |  |
|  | H_t1_ |  |  |  |  |  |  |  |  |  |  |  |
|  | H_t2_ |  |  |  |  |  |  |  |  |  |  |  |
| Female | Intercept | **-20.62899** | **-28.52272** | **-12.73525** |  | **0.33580** | **0.21734** | **0.45426** |  | **0.57394** | **0.46909** | **0.67880** |
|  | N_t0_ |  |  |  |  |  |  |  |  |  |  |  |
|  | N_t1_ |  |  |  |  |  |  |  |  |  |  |  |
|  | N_t2_ |  |  |  |  |  |  |  |  |  |  |  |
|  | D | **0.00097** | **0.00061** | **0.00133** |  |  |  |  |  |  |  |  |
|  | D^2^ | **-1.09e-08** | **-1.50e-08** | **-6.93e-09** |  |  |  |  |  |  |  |  |
|  | H_t0_ |  |  |  |  | **0.06668** | **0.05491** | **0.07846** |  | **0.15876** | **0.13620** | **0.18131** |
|  | H_t1_ |  |  |  |  |  |  |  |  | -0.01196 | -0.02662 | 0.00270 |
|  | H_t2_ |  |  |  |  |  |  |  |  | **-0.13166** | **-0.14627** | **-0.11705** |

D - Human density; H_t0_ – Human hunting pressure; H_t1_ – Human hunting pressure (lag 1); H_t2_ – Human hunting pressure (lag 2); N_t0_ – NDVI; N_t1_ - NDVI (lag 1); N_t2_ - NDVI (lag 2)

**Table S11** Estimated coefficients from substantially supported (ΔAICc <2) models to explain cougar-human conflict in the Vancouver Island region, British Columbia as a function of predictor variables. Variables for which estimates did not overlap zero are given in bold.

| **Cougar sex** | **Variable** | ***β*** | **90% CI** |  |  | ***β*** | **90% CI** |  |  | ***β*** | **90% CI** |  |
| --- | --- | --- | --- | --- | --- | --- | --- | --- | --- | --- | --- | --- |
| Male | Intercept | 0.30389 | -0.34596 | 0.95375 |  | 0.03933 | -0.64608 | 0.72473 |  |  |  |  |
|  | N_t0_ |  |  |  |  |  |  |  |  |  |  |  |
|  | N_t1_ |  |  |  |  |  |  |  |  |  |  |  |
|  | N_t2_ |  |  |  |  |  |  |  |  |  |  |  |
|  | D |  |  |  |  |  |  |  |  |  |  |  |
|  | D^2^ |  |  |  |  |  |  |  |  |  |  |  |
|  | H_t0_ |  |  |  |  | **0.04546** | **0.02023** | **0.07069** |  |  |  |  |
|  | H_t1_ | **0.06786** | **0.02409** | **0.11164** |  | **0.06183** | **0.02326** | **0.10040** |  |  |  |  |
|  | H_t2_ | **0.08378** | **0.06470** | **0.10287** |  | **0.07231** | **0.05550** | **0.08912** |  |  |  |  |
| Female | Intercept | 0.26978 | -0.24533 | 0.78489 |  | **0.45956** | **0.15153** | **0.76758** |  |  |  |  |
|  | N_t0_ |  |  |  |  | **-0.00004** | **-0.00005** | **-0.00003** |  |  |  |  |
|  | N_t1_ |  |  |  |  |  |  |  |  |  |  |  |
|  | N_t2_ |  |  |  |  |  |  |  |  |  |  |  |
|  | D |  |  |  |  |  |  |  |  |  |  |  |
|  | D^2^ |  |  |  |  |  |  |  |  |  |  |  |
|  | H_t0_ | **0.19890** | **0.12787** | **0.26993** |  | **0.20236** | **0.15472** | **0.25001** |  |  |  |  |
|  | H_t1_ |  |  |  |  |  |  |  |  |  |  |  |
|  | H_t2_ |  |  |  |  |  |  |  |  |  |  |  |

D - Human density; H_t0_ – Human hunting pressure; H_t1_ – Human hunting pressure (lag 1); H_t2_ – Human hunting pressure (lag 2); N_t0_ – NDVI; N_t1_ - NDVI (lag 1); N_t2_ - NDVI (lag 2)
